# Unexpected allergic reactions to food, a prospective study

**DOI:** 10.1186/2045-7022-5-S3-P149

**Published:** 2015-03-30

**Authors:** Anouska Michelsen-Huisman, Harmieke Os-Medendorp, Astrid Versluis, Astrid Kruizinga, Jacqueline Castenmiller, Hub Noteborn, Geert Houben, André Knulst

**Affiliations:** 1 UMC Utrecht, Utrecht, The Netherlands; 2 Netherlands Organisation for Applied Scientific Research (TNO), Zeist, The Netherlands; 3 Netherlands Food and Consumer Product Safety Authority (NVWA), Utrecht, The Netherlands

## Background

Severe reactions and even fatalities occur in patients with food allergy, due to unexpected allergen exposure, but frequency data are scarce. The aim of this study is to investigate the frequency, severity and causes of unexpected allergic reactions to food in adults diagnosed with food allergy.

## Methods

A prospective cohort study is carried out starting January 2012. A total of 180 adult patients with a physician-confirmed diagnosis of food allergy will be included and followed during one year. Outcome measures are frequency, severity and causes of unexpected reactions. Participants complete an online questionnaire after every unexpected reaction and send in the culprit product including the food label. The product is analyzed for suspected allergens.

## Results

Until now 152 patients (mean age=36 years (range 19-71), 70% female) have been included. The mean number of foods for which the patients are allergic was 5.5 (range 1-17). A total of 118 unexpected reactions were reported during the mean follow-up period of 9.7 months. Ninety-seven patients (64%) had no reactions, twenty-nine (19%) reported one reaction and twenty-six (17%) patients reported more than one reaction (range 2-11). Severity of these reactions was Muller 0: 18%, Muller I: 26%, Muller II: 27%, Muller III: 23% and Muller IV: 6%.

Preliminary results show that 44% (n=52) of the unexpected reactions were caused by pre-packaged products, 23% (n=27) by composite meals outdoors, for example at a friends' or relatives home, at school or in a restaurant, 17% (n=20) by fresh products, 8% (n=9) by composite meals at home and 8% (n=10) of the reactions took place abroad.

Main causes of unexpected reactions were labelling issues and risk taking behaviour of some patients. Forty-seven products were sent in to be analyzed. In one third of the products analyzed, the suspected allergen was detected.

## Conclusions

36% of the participants reported at least one unexpected allergic reaction. The mean number of reactions was 1 per person per year; reactions were mostly moderate to severe. Most reactions occurred after consuming pre-packaged foods. Labelling issues were frequently involved.

